# Microstructure characterization and corrosion resistance properties of Pb-Sb alloys for lead acid battery spine produced by different casting methods

**DOI:** 10.1371/journal.pone.0195224

**Published:** 2018-04-18

**Authors:** Asiful H. Seikh, El-Sayed M. Sherif, Sohail M. A. Khan Mohammed, Muneer Baig, Mohammad Asif Alam, Nabeel Alharthi

**Affiliations:** 1 Centre of Excellence for Research in Engineering Materials (CEREM), King Saud University, Riyadh, Saudi Arabia; 2 Department of Mechanical and Industrial Engineering, Ryerson University, Toronto, Ontario, Canada; 3 Engineering Management Department, Prince Sultan University, Riyadh, Kingdom of Saudi Arabia; 4 Mechanical Engineering Department, College of Engineering, King Saud University, Riyadh, Saudi Arabia; Universidade Estadual de Campinas, BRAZIL

## Abstract

The aim of this study is to find out the microstructure, hardness, and corrosion resistance of Pb-5%Sb spine alloy. The alloy has been produced by high pressure die casting (HPDC), medium pressure die casting (AS) and low pressure die casting (GS) methods, respectively. The microstructure was characterized by using optical microscopy and scanning electron microscopy (SEM). The hardness was also reported. The corrosion resistance of the spines in 0.5M H_2_SO_4_ solution has been analyzed by measuring the weight loss, impedance spectroscopy and the potentiodynamic polarization techniques. It has been found that the spine produced by HPDC has defect-free fine grain structure resulting improvement in hardness and excellent corrosion resistance.

## Introduction

Lead acid batteries are the most widely used battery system in several applications [1]. The ability of lead batteries to supply high surge currents at relatively lost cost makes it attractive for use in several applications especially in automobiles, where high current is required for the motors to start [2]. Due to the soft nature of pure lead, lead antimony alloys are used in the preparation of lead battery grids [3]. Small amounts of elements like arsenic and selenium are usually added to the lead antimony alloys to improve the grain refinement, fluidity and age hardening of the grids. It has been found reported [3] that that the content of Sb in Pb-Sb alloys significantly increases the mechanical properties and electrochemical process of the positive plate of the battery. Consequently, the influence of Sb on electrochemical behaviour in Pb-Sb alloys has been widely studied during last few decades [4–7].

Pb-Sb alloys with antimony ranging from 1 to 12% are used widely in pumps and valves, radiation shielding and lining the walls to contain the radioactive sources [8]. Babic et al. [9] studied the electrochemical behaviour of Pb, Sb and Pb-Sb in sulfuric acid solution. They reported that as the Sb content increases, its oxidation takes place in the alloy with decrease in film resistance [9]. In the past, several researchers found that the microstructure and electrochemical properties depends on Sb content in Pb-Sb alloys [10,11]. However, Pb-Sb alloys with 1.5–3.5 wt.% Sb becomes brittle and tends to crack because of the dendritic structure [12]. Moreover, few researchers studied the electrochemical properties of Pb-Sb alloys relating to the phase structure of the alloys, which are composed of α-Pb crystals and eutectic phase [13]. It is evident that the electrochemical behaviour of Pb–Sb alloys depends on the electrochemical property of the internal galvanic couples, formed by the individual phases. The addition of alloying agent decreases the activation energy of the oxidation which may lead to increased contribution of grain boundary corrosion [14].

Osório and co-workers [15–18] have studied the electrochemical behaviour of Pb-Sb alloys in lead acid battery applications. Where, the effect of microstructural morphologies of Pb-6.6wt% Sb alloy on the corrosion resistance at different temperatures was evaluated. It was concluded that with increase in temperature, the corrosion resistance decreases. Osório et al. [16] also studied the comparison on Pb-1wt. %Sb alloy in 0.5 M NaCl and H_2_SO_4_ solutions. Rezaei [19] studied the effect of casting temperature of the electrodes on the change of the polarization potential for lead acid batteries. The impurities present in the Pb alloys penetrate into the mould with less content on the electrode surface at low temperatures which causes change in electrochemical behaviour of the grids. Consequently, the casting temperature is a very significant parameter in order to obtain desired electrochemical behaviour of the grids.

Nevertheless, to the authors’ knowledge, investigation of corrosion behaviour of Pb-Sb alloys prepared by different casting methods has not been addressed yet. In the present study, the microstructural characterization using optical microscopy has been investigated. In addition, the electrochemical corrosion behaviour of Pb- 5wt. % Sb alloy by three casting methods viz. HPDC, gravity (GS) and auto (AS) spine samples were evaluated. This is because these materials are widely used in lead acid battery industry for positive spine casting and our study will provide more information concerning the better life of these spines.

## Materials and methods

### Specimen preparation

**Fig 1:**
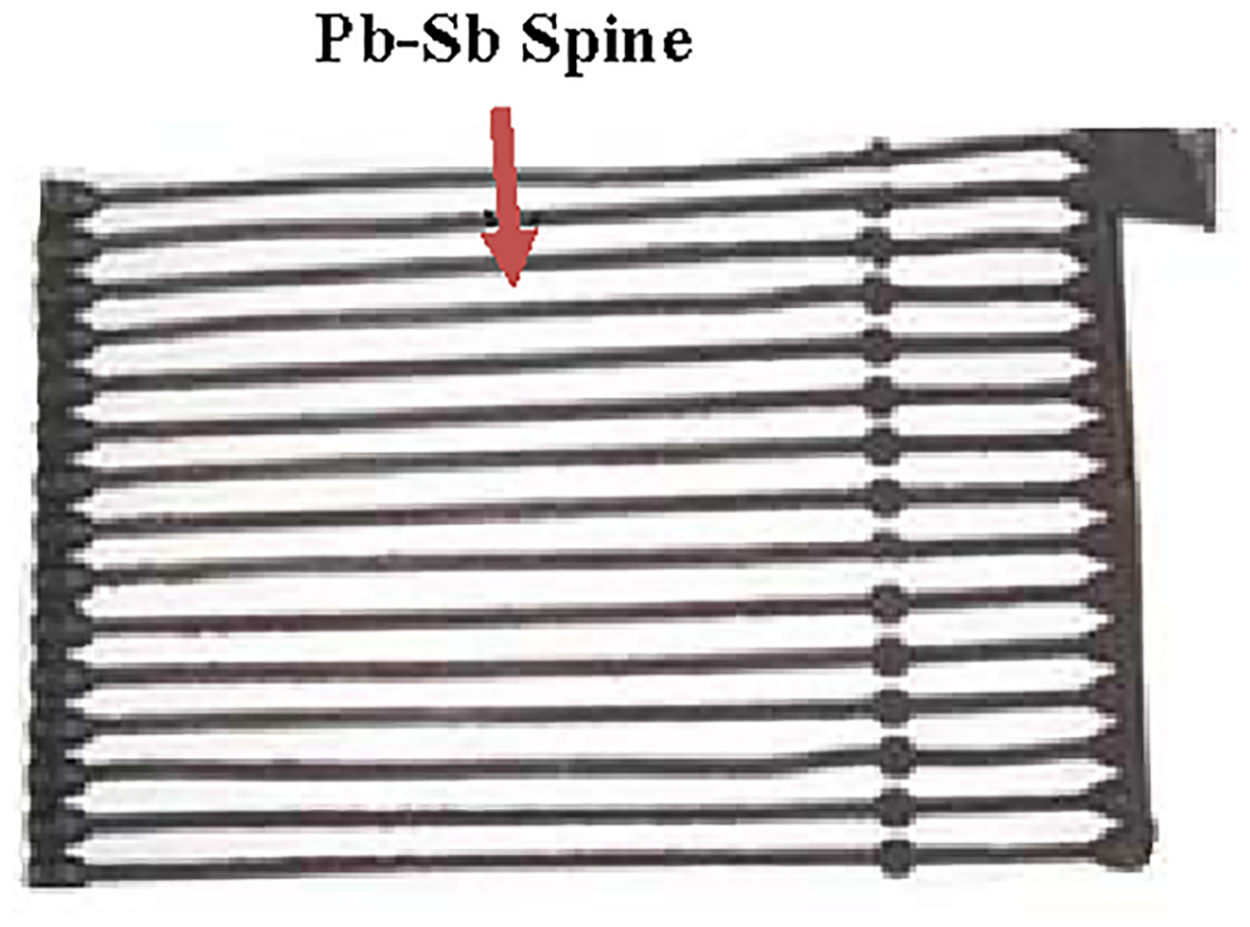
Pb-Sb spine and pasted positive plates used in lead acid batteries produced by casting methods.

Three grades of spines were produced by high, low, and very low pressures using HPDC, AS and GS casting methods, respectively. Hadi spine (HPDC) was prepared at a pressure of 11MPa whereas; auto spine (AS) was prepared at 9MPa and gravity spine (GS) at normal hand pressure of about 0.1MPa. More details on the casting processes can be found elsewhere [20]. The typical Pb–Sb grid is shown in Fig 1. The chemical composition of spines made of antimonial lead (5% Sb) is listed in Table 1. Sulphuric acid (H_2_SO_4_, 98%) was purchased from Merck and the used concentration, 0.5 M, was obtained by dilution.

**Table 1:** Chemical compositions of the investigated spine.

Sb	As	Sn	Cu	Se	Ca	Ni	Zn	Bi	Ag	Fe	Pb
5.0 ±0.2	0.15 ±0.20	0.15 ±0.20	0.04 ±0.01	0.005 max	0.002 max	0.005 max	0.002 max	0.030 max	0.010 max	0.005 max	Balance

### Optical microscopy (OM)

The exposed surface of the mounted specimens were grounded mechanically on the different grits of silicon carbide abrasive papers (from 200 up to 2000 grits) and polished on a Sylvet cloth using coarse and fine Geosyn- Grade I slurry of Al_2_O_3_. The surfaces of the spines were cleaned, washed by water followed by alcohol and dried in air. The specimens were then etched in a solution of 37.5 mL glacial acetic acid and 15 mL of H_2_O_2_. The microstructure of the etched specimens was investigated using an optical microscope and the photographs were collected using a camera was fitted to that microscope.

### Hardness testing

Buehler microhardness tester with a Vickers diamond tip was used to measure microhardness of three spine specimens. The measurements were performed at an indentation load of 100 gmf with a dwell time of 10 seconds. Prior to hardness measurements, the specimens were wet ground using emery papers of different grit sizes ranging from 220 to 2000 to obtain a mirror polished surface and the surface hardness measurements were then recorded. The measurements were triplicate and the average at each point has been recorded.

### Weight-loss

The loss in weight measurements were performed on rectangular coupons of area 6.8 cm^2^ prepared from three different spines of Pb-Sb alloys. The exposed area was polished extensively with sand papers, washed with distilled water and air dried followed by initial weight measurement (W_i_). The samples were immersed in an acid solution of 0.5 M H_2_SO_4_ at a constant temperature of 298K for 7 days. The solution was charged with constant voltage of 3V with parallel connection (45 A current max input). The weight loss measurements were recorded after the cleaning the specimen. The cleaning procedure includes cleaning with Mannitol solution for 30 mins and dried in an oven. The weight of the coupons for the three different spines were measured before immersion, W_i_ and after being immersed in the acid solution for 7 days, W_f_. The loss in weight (ΔW, gm.cm^−2^) and the corrosion rate (R_Corr_, gm/cm^2^/day) were calculated after the defined exposure time as follows [21]:

$${\text{R}}_{\text{Corr}}=\left({\text{W}}_{\text{i}}-{\text{W}}_{\text{f}}\right)/\text{A}.\text{t}$$ (1)

where, A is exposed area in cm^2^ which is 6.8, t is the exposure time in days. The corrosion rates of the spines were compared in terms of the weight-losses. All the weight loss measurements were carried out at room temperature. The weights were measured at least three times and the average loss in weight was considered. An average value of ±1.0% was observed as the maximum standard deviation in the observed weight loss.

### Electrochemical experiments

An Autolab system by Metrohm (PGSTAT20) was employed to carry out the electrochemical tests. The working electrode used for the electrochemical experiments was spine specimens with Ag/AgCl (E = + 0.222 V vs. saturated hydrogen electrode (SHE) at 25 °C) as a reference electrode and platinum foil as counter electrode. Square coupons of 1 cm length and 0.2 cm thickness were cut from three different spines as test specimens in the corrosion studies. The rear surface of the specimen was soldered with a copper wire to form an electrical connection, which acts as working electrode. The specimens were mounted in a glass holder after being embedded into two component epoxy resin. The exposed surface of 1 cm^2^ area was then wet polished with SiC abrasive papers to obtain 1000 grit. The polished surface was then air dried after washed in distilled water and acetone [22]. Preparation of electrolytic solution was made from analytical grade sulfuric acid and distilled water. The working electrodes were immersed in 0.5 M H_2_SO_4_ solution for 1 hour at open circuit potential (E_OCP_) until the potential become stable within ±1 mV. Electrochemical impedance spectroscopy were measured at E_OCP_ with applied 5 mV sinusoidal perturbations in the frequency range of 100 kHz ~ 0.1 Hz with 10 steps per decade. The potentiodynamic polarization was conducted by stepping the potential in the range of −250 mV to +250 mV against (Ag/AgCl from the value of the E_OCP_ with a scan rate of 1.67mV/s. For each experiment, freshly polished electrodes were used in a new portion of the electrolyte.

## Results and discussion

### Microstructures

**Fig 2:**
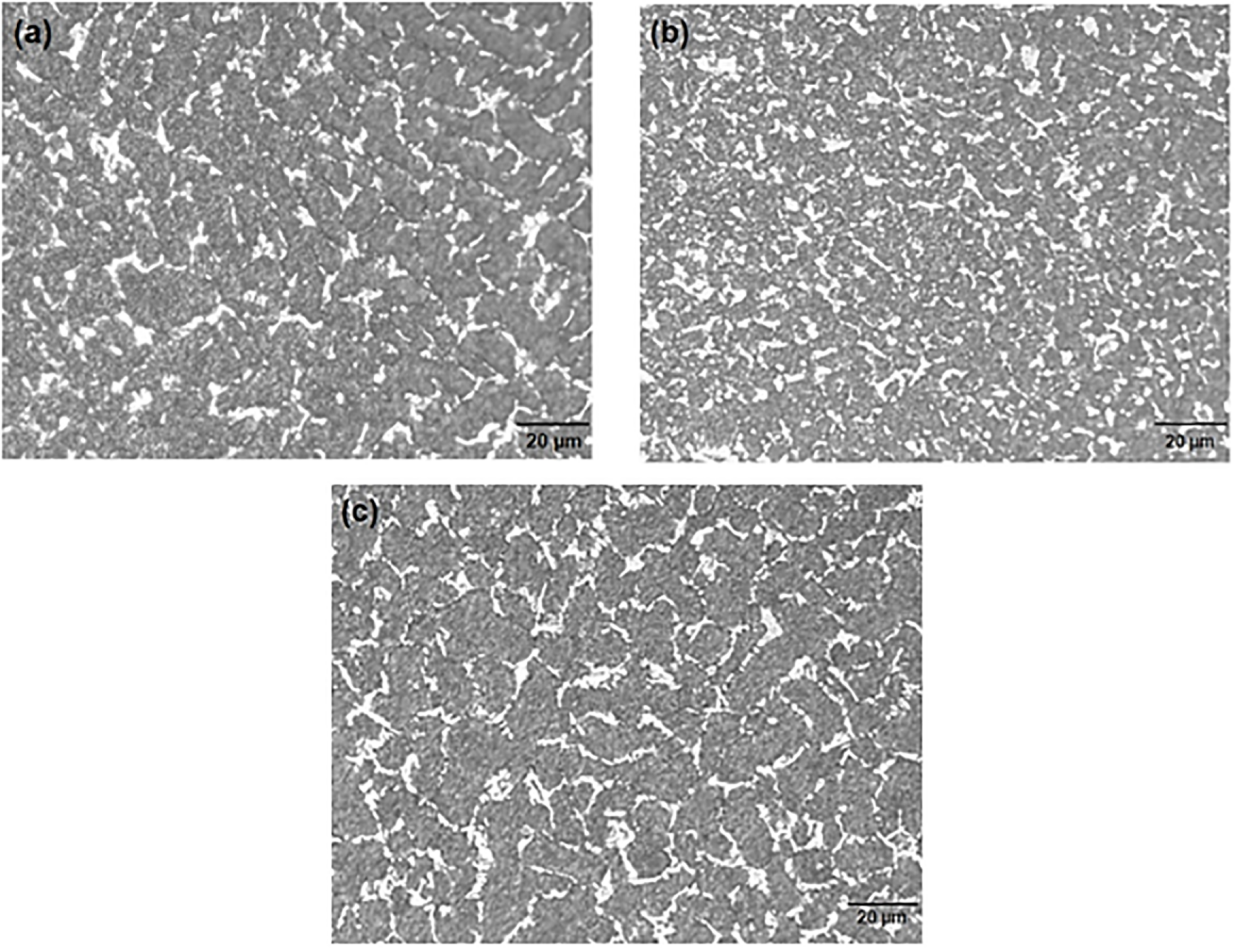
Optical micrograph of Pb-5%Sb alloy produced (a) high pressure (hadi, HPDC) casting (b) low pressure (auto (AS)) casting and (c) very low pressure (gravity (GS)) casting.

**Fig 3:**
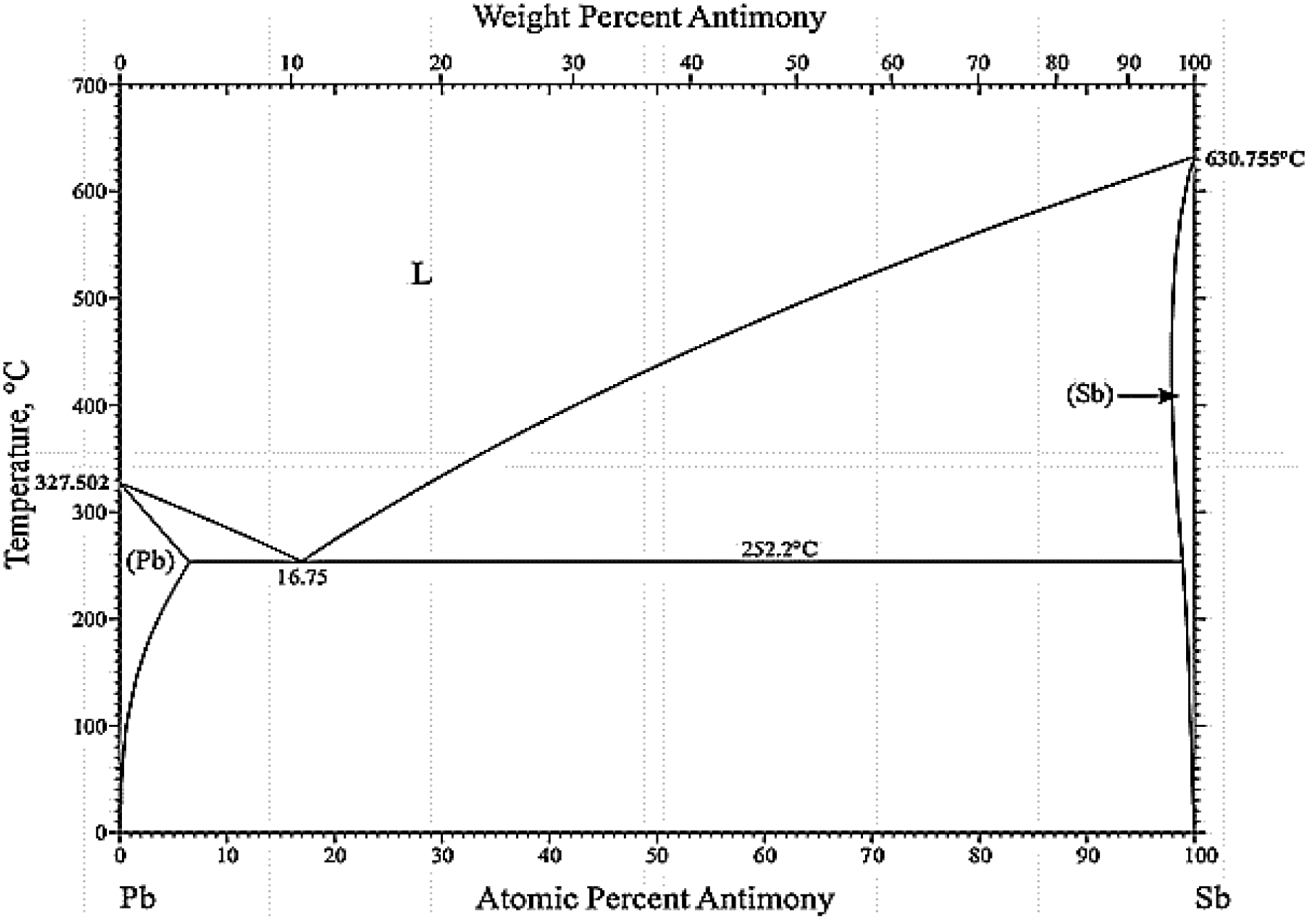
The Pb–Sb phase diagram.

Fig 2 shows the optical microscopy (OM) image for the microstructure of solidified Pb-5 wt. % Sb alloy spines of HPDC, AS and GS castings. It is evident from the Fig 2 that the microstructure mainly consists of a dendritic Pb rich matrix with a lamellar eutectic mixture in the interdendritic regions constituted of α, and a Sb-rich β-phase. The Pb-rich matrix is represented by white regions while the intercellular area is represented by dark regions. The eutectic fraction increases with the solute (Sb) content in the alloy (Pb-Sb) as shown in the phase diagram of Fig 3, which has been extracted from the work that has done by H. Okamoto [23].

**Fig 4:**
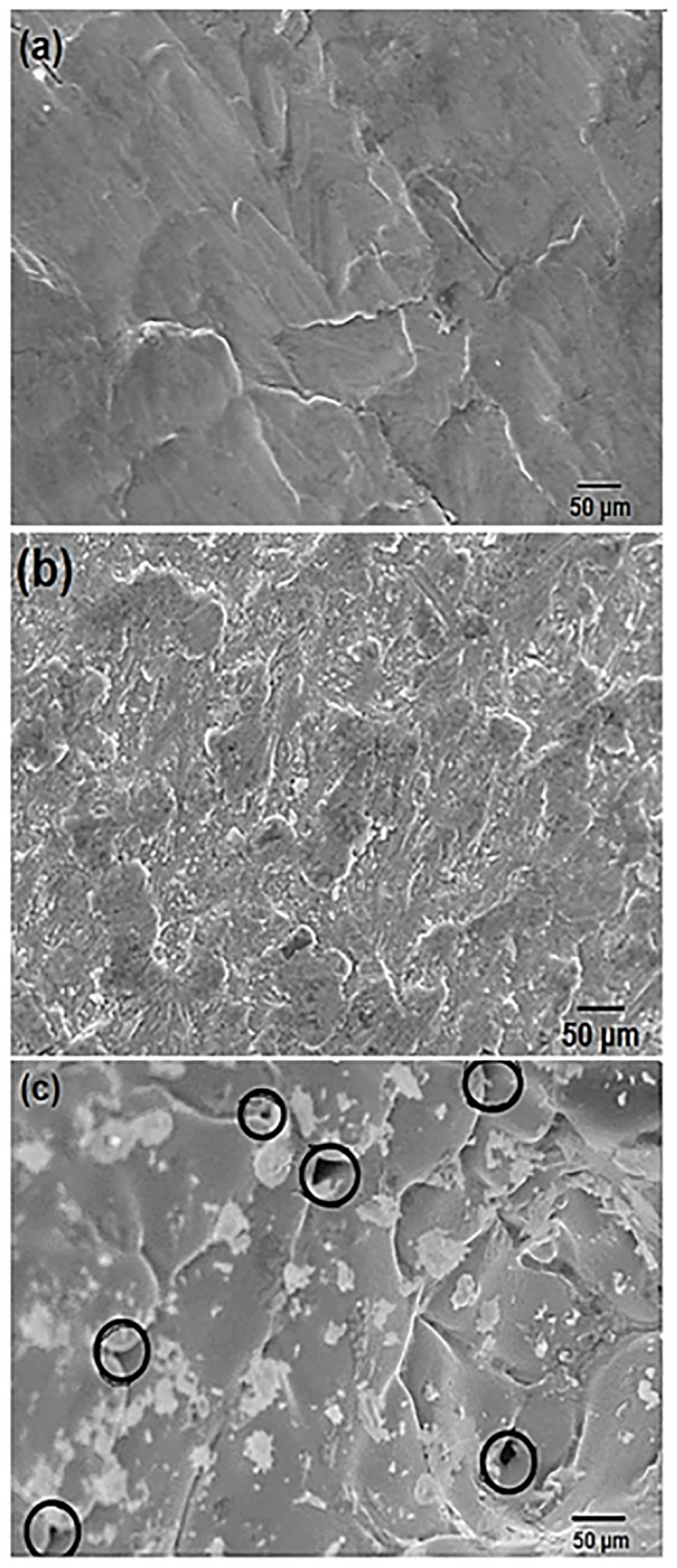
SEM micrographs of Pb-5%Sb alloy produced (a) high pressure (HPDC) casting (b) low pressure (AS) casting and (c) very low pressure (GS) casting.

The SEM micrographs of the as cast Pb-5%Sb alloys are depicted in Fig 4. It is seen for HS casting that the grain size is fine with grains oriented in uni-direction. In case of AS casting, the grain size is mixed i.e. coarse and fine and grain orientation is random whereas inconsistence grain size with random grain orientation is found for GS casting. For HPDC casting, no voids are presents on the surface whereas in AS casting voids with small dark spots and bigger voids are presents in GS casting. More information about the microstructure on different grades of Pb-Sb alloy spines can be seen from S1, S2, S3, S4 and S5 Figs.

### Hardness

The hardness value for the high pressure casting spine (HPDC) have uniform micro hardness of 24.3±0.5 HV throughout the sample and that of auto casting spine (AS) gives non-uniform microhardness varying from 21.5 to 22.4 HV. Whereas, in case of gravity casting (GS) the microhardness values is lower, varying from 17.5 to 18.2 HV. S1 Table shows the effect of increasing the percentage of Sb on the values of hardness recorded for different Pb-Sb alloys.

### Corrosion resistance behavior

#### Weight-loss measurements

The weight-loss and corrosion rates of the three different spines were calculated and presented in Table 2. It is seen from Table 2 that after 7 days of exposure, the weight-loss and corrosion rate of low pressure casting spine (GS) is more than that of high pressure casting spines (HPDC and AS). This confirms that the corrosion resistance for the investigated spines decreased as per the following order; HPDC > AS > GS. It has been observed that HPDC has smaller grain structure than AS and GS resulting highest grain boundaries. As a result of high grain boundaries, corrosion has to suffer high path and resulting low corrosion rate.

**Table 2:** Corrosion data obtained for the three different spines by weight-loss method.

Alloy type	W_i_ (gm)	W_f_ (gm)	ΔW (gm/cm^2^)	R_Corr_ (gm/cm^2^/day)
**HPDC**	372	320	7.65	1.09
**AS**	401	333	10	1.43
**GS**	346	255	13.38	1.91

#### Polarization measurements

**Fig 5:**
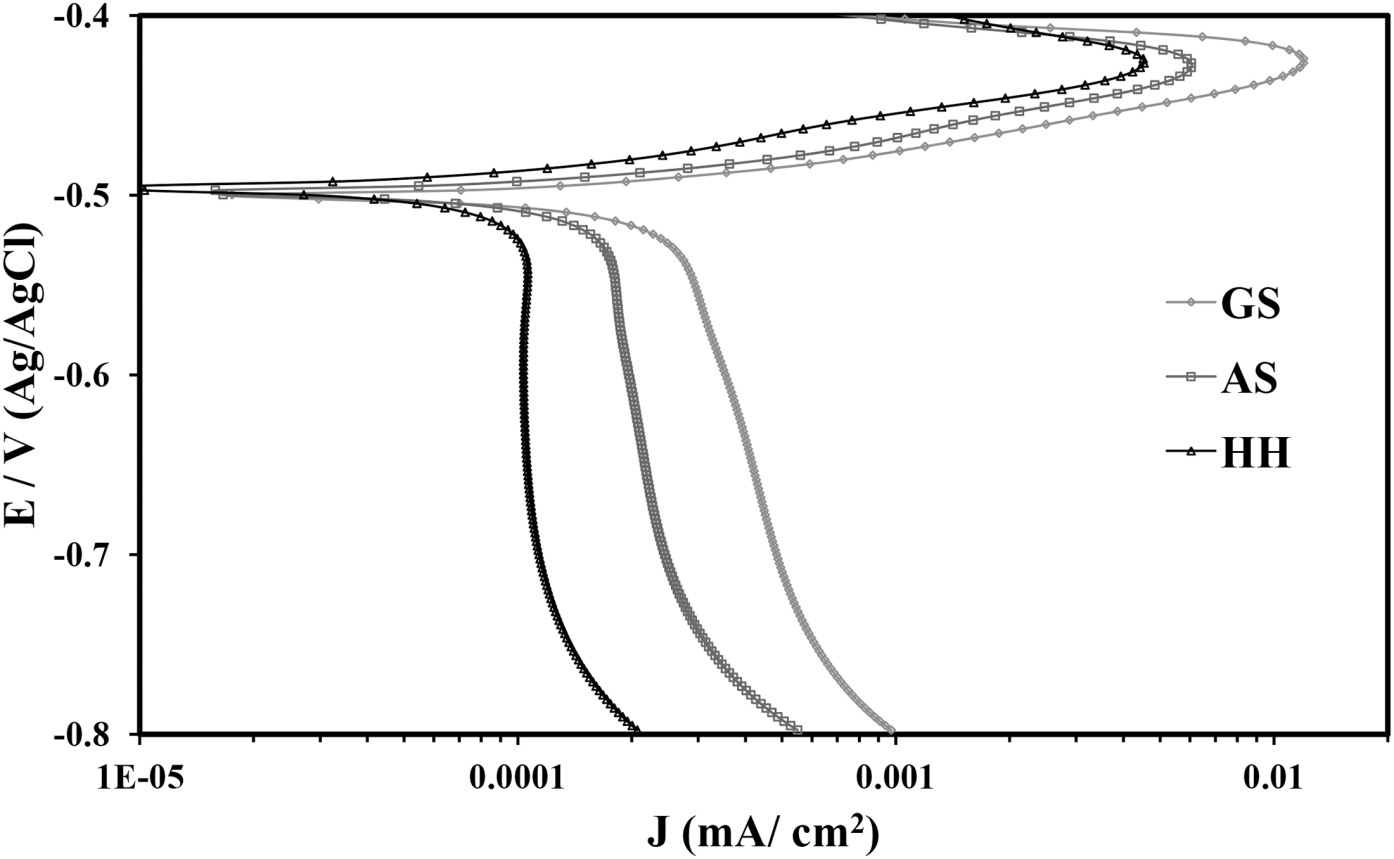
Potentiodynamic polarization curves for different Pb-5%Sb alloy in 0.5 M H_2_SO_4_ at room temperature.

The electrochemical performance of the different casting spines of Pb-5% Sb alloy was investigated after exposing in 0.5 M H_2_SO_4_ solution for 1 h. The potentiodynamic polarization curves for HPDC, AS, and GS are shown in Fig 5, from which the corrosion data were obtained by Tafel extrapolation technique and the values are listed in Table 3. The corrosion current density (j_Corr_) was obtained from the intersection of cathodic and anodic slopes of the polarization curves. The effect of increasing the percentage of Sb on the values of hardness recorded for different Pb-Sb alloys can be seen from S2 Table.

**Table 3:** Potentiodynamic polarization data obtained for the different spines immersed in 0.5 M H_2_SO_4_ for 1 h.

Tafel Data					Linear Polarization Data
Alloy type	β_a_ / mVdec^-1^	β_c_ / mVdec^-1^	E_Corr_ / mV	j_Corr_, μAcm^-2^	R_p /_ Ωcm^2^
**HPDC**	56.83	64.52	-491	55	167.7
**AS**	26.87	33.09	-502	94	68.5
**GS**	21.56	28.97	-512	140	38.4

The experimental polarization curves of the spines of the different casting conditions carried out in 0.5 M H_2_SO_4_ solutions show similar trend. The cathodic reaction is the hydrogen evolution reaction because of the sulfuric acid solution, while the anodic reaction is the dissolution of the alloy causing the increase of current in the anodic side. The corrosion potential (E_OCP_) was found to be in between −0.491 V and −0.512 V (Ag/AgCl). It was observed that the value of E_OCP_ increases from -0.491 V (Ag/AgCl) towards the nobler side along with the dendritic spacing i.e., grain size of the spines. The decrease of current we have seen on the anodic curves after the active dissolution might have been resulted from the formation of a top layer of lead oxide(s) such as PbO and/or PbO_2_ or even lead sulfate, PbSO_4_. It is also found that the corrosion current density, which is directly proportional to the corrosion rate, of high pressure casting spines (HPDC and AS) is lower than that of low pressure (GS) casting spine. This indicates that the corrosion resistances of HPDC and AS spines are higher than GS spine at the same conditions, i.e. the corrosion resistance decreases in the order HPDC > AS > GS and its behavior agrees with the data obtained from the weight-loss.

#### Electrochemical impedance spectroscopy (EIS) studies

**Fig 6:**
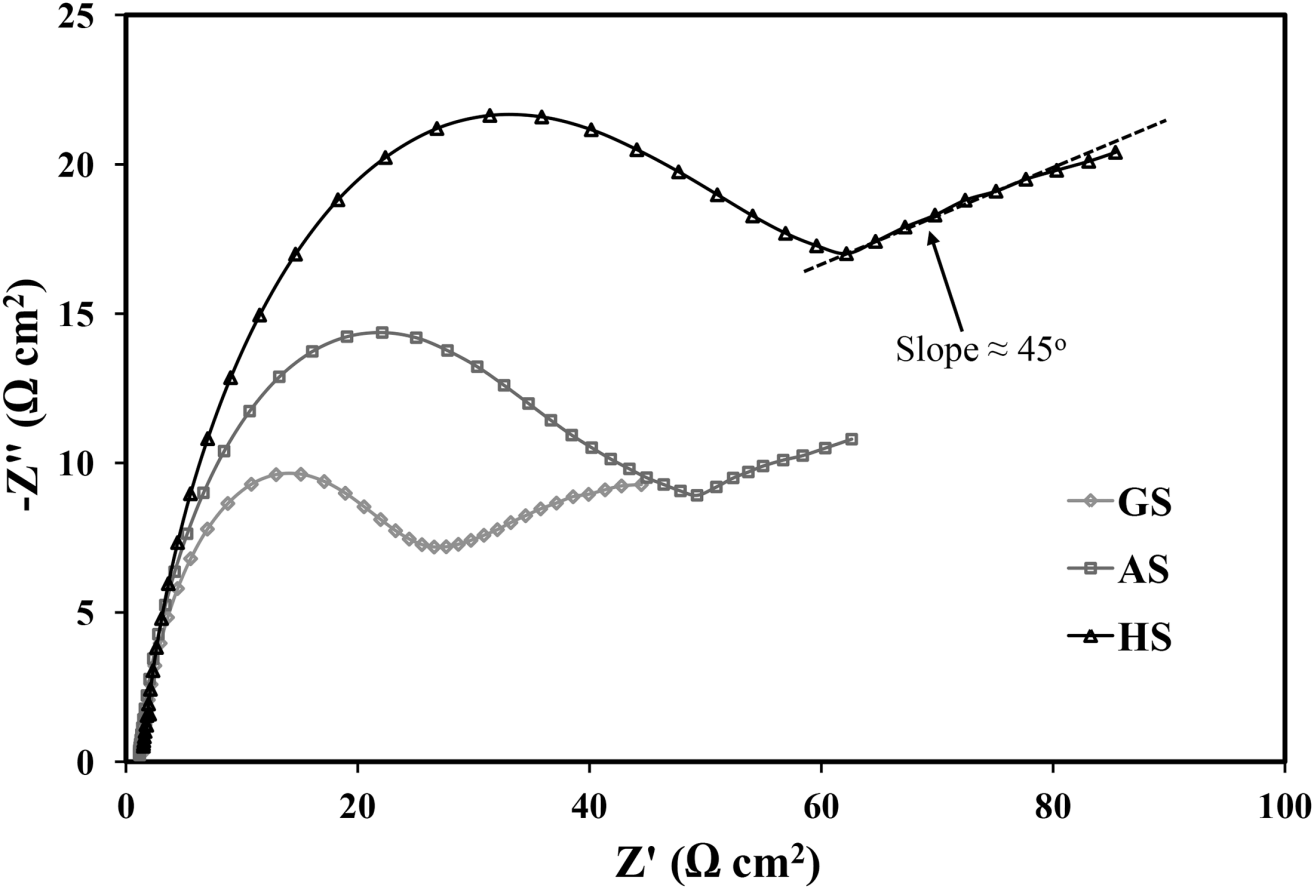
Nyquist plots for different Pb-5%Sb alloy in 0.5 M H_2_SO_4_ at room temperature.

**Fig 7:**
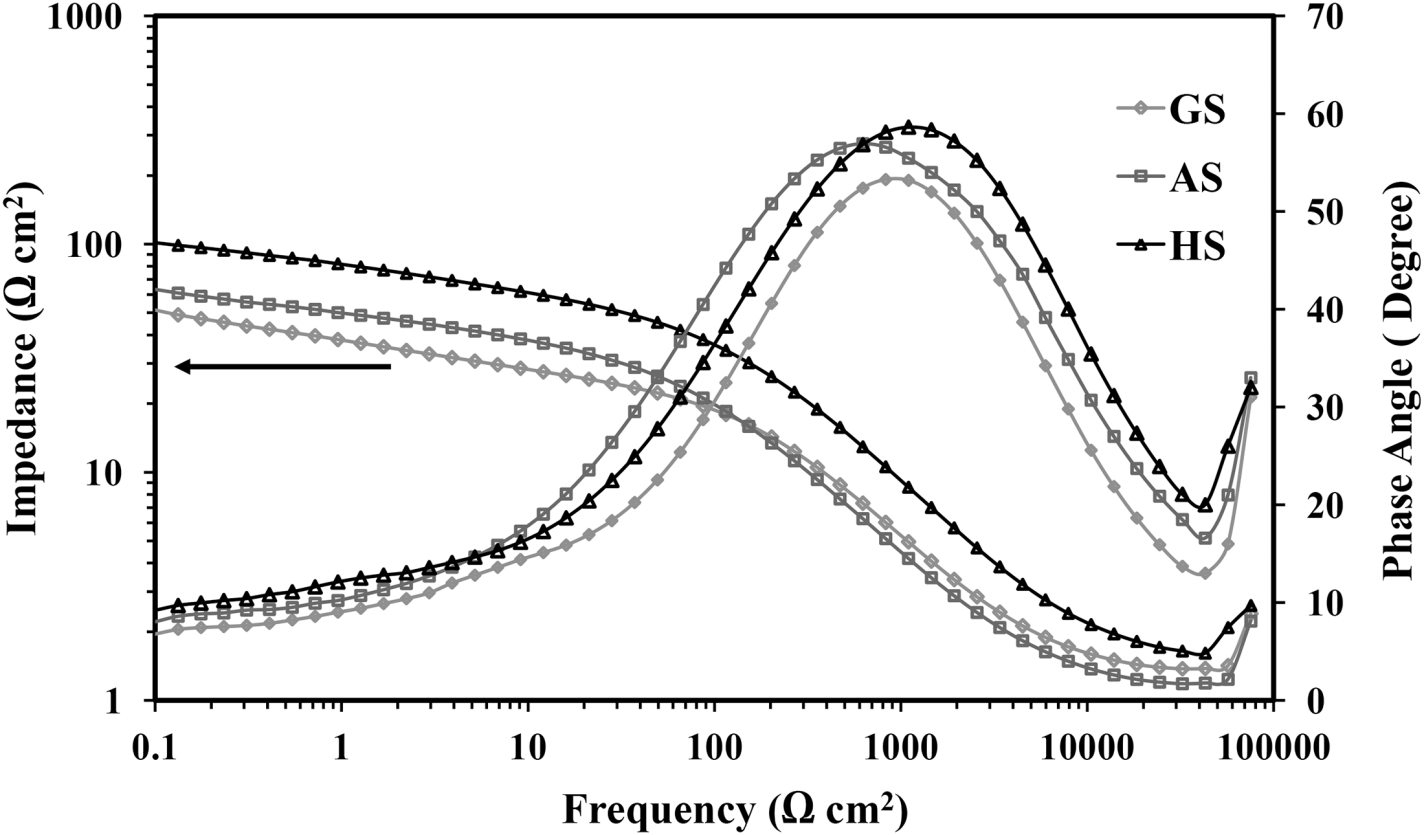
Bode plots for different Pb-5%Sb alloy in 0.5 M H_2_SO_4_ at room temperature.

Figs 6 and 7 show the Nyquist, Bode impedance of the interface and Bode-phase plots for HPDC, AS, and GS after one hour of exposure time in 0.5 M H_2_SO_4_. Bode-phase diagram showed two time constants related to the corrosion kinetics of the Pb-5 wt.% Sb alloy. The first time constant at a frequency range from 10^4^ to 10^5^ Hz related to the reaction between the acid solution and the Sb-rich phase in the interdendritic region. Whereas, the second time constant in the 10^2^ to 10^0^ frequency range associates with the Pb-rich matrix, which is clearly shown for all the alloys, as represented in Fig 6.

The results of Nyquist plots presented in Fig 6 exhibits a capacitive arc increasing between 10^5^ Hz and 1 Hz. However, for frequencies lower than 1 Hz, the capacitive arc increases with a slope of 45°. Similar behavior has been reported in the past for Pb-Sb and Pb-Sn alloys [15–18]. This indicates that an oxide film has formed on the surface, which shows good corrosion resistance of Pb-Sb alloys. It is seen from Fig 6 also that the diameter of the semicircle obtained for the different spines recorded wider diameter for HPDC than AS and GS. It is well known that the wider the diameter of the semicircle is the more corrosion resistance obtained for the materials under investigation. From this point of view, HPDC shows higher corrosion resistance than AS, while GS has the lowest corrosion resistance. The Bode impedance of the interface (|Z|) and the Bode-phase (ϕ) plots presented in Fig 7 revealed that the values of the |Z| recorded higher values for HPDC alloy across the whole range of the investigated frequency; these values decrease for AS then GS, respectively. The same trend were recorded with the values of ϕ, where the maximum values of ϕ were found to be for HPDC alloy followed by AS and finally GS.

**Fig 8:**
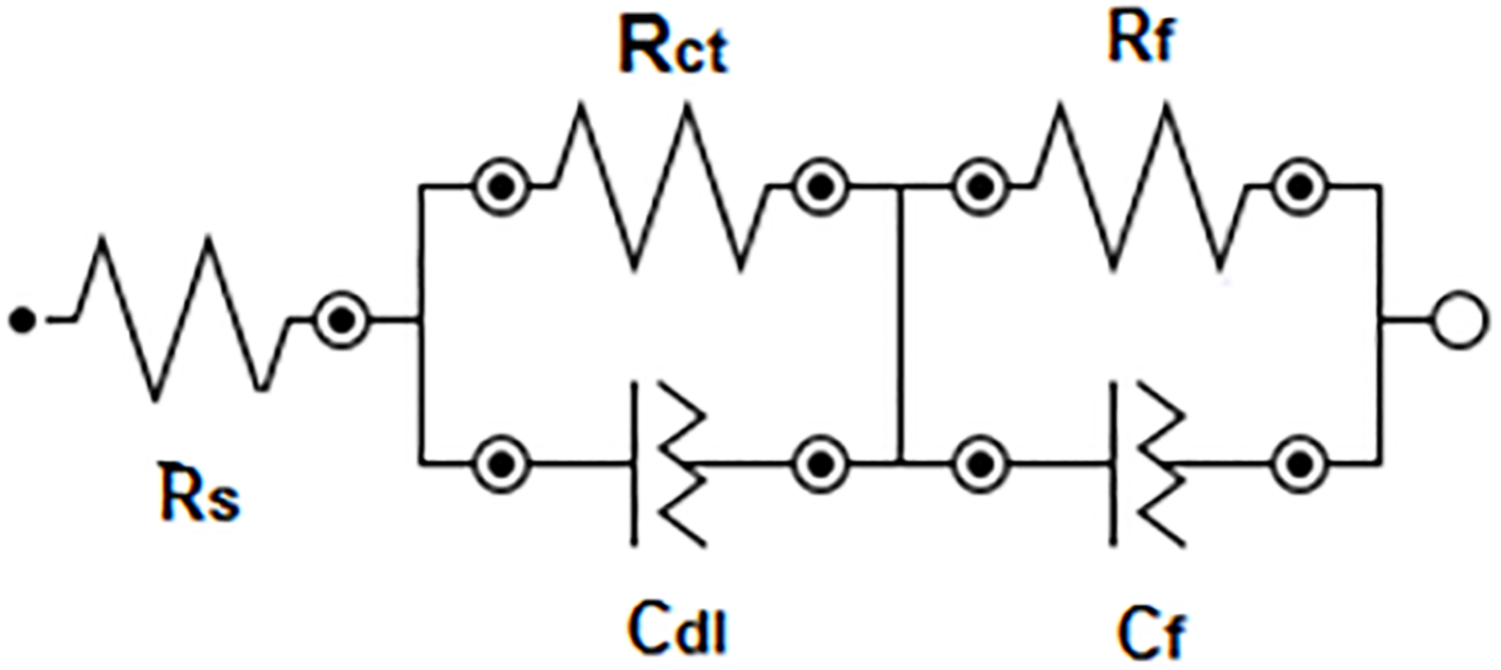
Equivalent circuit fitting for the EIS data presented in Fig 6.

An equivalent circuit model has been modeled to present the quantitative analysis of EIS results, as reported in previous studies [15–18]. Fig 8 shows the recommended equivalent circuit used in this analysis to fit the experimental data. The impedance parameters obtained by the ZView^®^ software are shown in Table 4. The physical elements of the equivalent circuit are in accordance with the models presented in the literature [15–18]. The parameters evaluated during the study include the electrolyte resistance (Rs), the charge transfer resistance (R_ct_), and the polarization resistance due to the participation of adsorbed intermediates (R_f_). The pseudo double layer constant (capacitive elements CPE_dl_ (C_dl_)), which represents the double-layer capacitance and CPE_f_ (C_f_), which indicates capacitance associated with polarization resistance R_f_, are substituted with constant phase elements (CPEs) to obtain an accurate curve fit which matches the experimental data. The values of n_1_ and n_2_ are well known to be varying between −1 and 1. Z_CPE_ = [C(jω)^n^]^−1^ denotes the impedance of a constant phase element [15–18, 24–26] and are correlated with the phase angle; where, C is the capacitance; j is the current; ω is the frequency and −1 ≤ n ≤1. It has been reported [25] that the value of n is associated with the non-uniform distribution of current due to the presence of some defects or roughness for the surface.

**Table 4:** EIS parameters obtained for HPDC, AS, and GS immersed in 0.5M H_2_SO_4_ for 1 h.

Alloy Type	Kinetic EIS parameters						
	R_S_ / Ω cm^2^	CPE_dl_		R_ct_ / Ω cm^2^	CPE_f_		R_f_ / Ω cm^2^
		Yc_dl_/F cm^-2^	n1		Yc_f_/ F cm^-2^	n2	
**HPDC**	1.39	0.000135	0.79	56	0.00066	0.65	57
**AS**	0.8	0.000119	0.80	34	0.00157	0.47	44
**GS**	1.08	0.000100	0.91	18	0.0010	0.49	41

Various parameters represent the corrosion resistance of the alloy. However, R_ct_, which is inversely proportional to j_Corr_ explicate the corrosion resistance of the alloy. From the impedance parameters in Table 4, it is seen that the value of C_f_ is always lower than C_dl_. The decrease in the capacitance is attributed to the increase in passive layer [24] and decrease in the dielectric constant of oxide film caused due to alteration in the ratio of electrolytic volume and oxide film [25–28]. The double layer formation on the surface of the sample is represented by the capacitance C_dl_. The higher value of C_dl_ demonstrates better corrosion resistance. From the EIS parameters in Table 4, it can be seen that spines produced by high pressure casting (HPDC and AS) have higher capacitance C_dl_ and higher polarization resistance than the spine produced by low pressure casting (GS). This could be due to the fine dendrite arm spacing i.e., fine grain size in HPDC and AS compared to coarse grain size in GS. It can be concluded from both experimental EIS results and potentiodynamic polarization curve fits that the fine structure of the dendritic morphology reveals better corrosion resistance compared to the coarse dendritic structure. The EIS data thus confirm the results obtained by weight-loss and polarization that the corrosion resistance of the investigated spines decreases in as per the following order; HPDC > AS > GS.

#### Corrosion mechanisms

The corrosion mechanism of three different spines (Pb-Sb alloy) produced by different casting methods, structures of spines and corrosion results were analyzed. As mentioned before the microstructure of the as cast Pb-Sb alloy is dendritic, mostly formed by α—phase (less noble) and the interdendritic is composed of lamellar eutectic morphology, which is mixture of α- and β-phases. The corrosion response happens predominantly in Pb-rich dendritic matrix which is more susceptible to corrosion compared to the inactive interdendritic region (Sb-rich nobler region). It has been reported that for a non-dilute Pb–Sb alloy (Pb–6.6 wt. % Sb), the finer dendritic region have strong corrosion resistance than coarse dendritic structure [11]. The dendritic morphology is composed of Sb rich areas located in the lamellar eutectic blend. The antimony rich lamellae encrust the lead rich region to obtain the fine dendritic spacing. This is due to the extensive dispersion of the eutectic mixture which leads to protection of the Pb-rich matrix. In sharp contrast, the matrix which exposes more lead will have a relatively coarse dendritic structure and thus escalates the corrosion behavior.

The work under investigation emphasizes on the role of the grain size on the electrochemical behavior of the Pb-Sb alloy battery spines produced by different casting methods. In this study, the potential range selected was low, so there was no formation of PbO or PbO_2_. The corrosion resistance investigation in this work is differing from the corrosion of Pb electrode, which progress at higher potentials by the formation of PbO/PbO_2_ [29,30]. As it is well known that the E_OCP_ of Pb-Sb alloy is higher than that of pure Pb because of the faster self-discharges associated with the lower hydrogen over-voltage on Sb [28–30]. At a potential above the E_OCP_ of Pb, the anodic oxidation of Pb takes place and Pb^2+^ ions are formed or reduced along with some hydrogen evolution. As the solubility of PbSO_4_ in 0.5M H_2_SO_4_ is relatively high, the electrode surface develops only some PbSO_4_ crystals. After prolonged polarization, a semi-permeable membrane of PbSO_4_ is formed that passivates the electrode surface. On the other hand, not any PbO, which is a stronger passivation, Sb ions or mixed Pb–Sb oxides are not being formed on the surface of the electrode [29–31]. It may be referred to that the rate for anodic oxidation from Pb to Pb^2+^ ions at a potential of −0.8V may be not the procedure that determines the corrosion rates of lead in H_2_SO_4_. Furthermore, the anodic oxidation of lead, during potentials over −0.40V, an alternate anodic procedure starts in which Sb oxidation to Sb^3+^ and Sb^5+^ may be included. These techniques were efficiently discussed by Pavlov et al [29–31].

In view of the spines analyzed in this investigation, it might have been interesting to see that the corrosion rate declines with reducing dendritic arm spacing i.e. a finer structure spine gives better corrosion resistance. The results also predict that the operational conditions of the casting system effects the as cast microstructure of Pb-Sb spines as well as the corrosion resistance behavior. In order to produce as-cast Pb–Sb spines with better corrosion behavior, the manufacturer of lead acid batteries must go for appropriate manufacturing process.

## Conclusions

The electrochemical behavior of lead-acid battery spines (Pb-5%wt Sb alloy) were investigated in 0.5M H_2_SO_4_ solution employing the loss in weight, potentiodynamic polarization and impedance spectroscopy measurements. These Pb-Sb spines were produced by three different casting methods. The metallography results indicated that the microstructures of the three spines composed of dendritic structures with Sb-rich regions that are located in the lamellar eutectic mixture. The experimental corrosion measurements revealed that the corrosion resistance of the spines resulted from the finer dendritic arrays rather than the coarser dendritic structures. The finer dendritic spacings tend to yield Pb-rich phase more efficiently and this is due to the more extensive distribution of the eutectic mixture, which in turn leads to higher protection for the Pb-rich matrix. Results together confirmed that the three spines have shown high corrosion resistance in 0.5 M H_2_SO_4_ and the corrosion resistance increases according to the following order; GS < AS < HPDC.

## Supporting information

S1 Table Vickers hardness values for the different Pb-Sb alloys. (DOCX)

S2 Table Corrosion parameters obtained for the different Pb-Sb alloys. (DOCX)

S1 Fig The microstructure of the pure lead (0%Sb). (TIF)

S2 Fig The microstructure of the Pb-1%Sb alloy. (TIF)

S3 Fig The microstructure of the Pb-2.5%Sb alloy. (TIF)

S4 Fig The microstructure of the Pb-5%Sb alloy. (TIF)

S5 Fig The microstructure of the Pb-9%Sb alloy. (TIF)
