# Correction: Microstructure characterization and corrosion resistance properties of Pb-Sb alloys for lead acid battery spine produced by different casting methods

**DOI:** 10.1371/journal.pone.0197227

**Published:** 2018-05-08

**Authors:** The PLOS ONE Staff

The second affiliation for the author El-Sayed M. Sherif is not indicated. This author is also affiliated with: Electrochemistry and Corrosion Laboratory, Department of Physical Chemistry, National Research Centre, El-Behoth St. 33, Dokki, Cairo 12622, Egypt. The publisher apologizes for this error.
