# Supplementary material for: Microstructure characterization and corrosion resistance properties of Pb-Sb alloys for lead acid battery spine produced by different casting methods
Source: PLoS One. 2018 Apr 18;13(4):e0195224. doi: 10.1371/journal.pone.0195224 (PMC5905994; doi:10.1371/journal.pone.0195224)
Supplement: S1 Table — (DOCX) [file pone.0195224.s001.docx]

| **Alloy** | **Vickers Hardness (HV)** |
| --- | --- |
| Pb-0%Sb | 8.4 |
| Pb-1%Sb | 9.2 |
| Pb-2.5%Sb | 13.7 |
| Pb-5 % Sb | 17.8 |
| Pb-9% Sb | 22.8 |
