# Supplementary material for: Microstructure characterization and corrosion resistance properties of Pb-Sb alloys for lead acid battery spine produced by different casting methods
Source: PLoS One. 2018 Apr 18;13(4):e0195224. doi: 10.1371/journal.pone.0195224 (PMC5905994; doi:10.1371/journal.pone.0195224)
Supplement: S2 Table — (DOCX) [file pone.0195224.s002.docx]

| **Sample** | **j _Corr_ (A/cm^2^)** | **E_Corr_ (V)** | **R_Corr_ (mm/year)** |
| --- | --- | --- | --- |
| Pb-0%Sb | 1.30 X10^-6^ | -0.964 | 4.91 X 10^-1^ |
| Pb-1%Sb | 9.48 X10^-6^ | -0.964 | 3.81 X 10^-1^ |
| Pb-2.5%Sb | 1.46 X10^-5^ | -0.962 | 2.76 X 10^-1^ |
| Pb-5 % Sb | 9.35 X10^-6^ | -0.959 | 1.80 X 10^-1^ |
| Pb-9% Sb | 3.82 X10^-6^ | -0.952 | 1.14 X 10^-2^ |
