# Supplementary figures and images for: Microstructure characterization and corrosion resistance properties of Pb-Sb alloys for lead acid battery spine produced by different casting methods
Source: PLoS One. 2018 Apr 18;13(4):e0195224. doi: 10.1371/journal.pone.0195224 (PMC5905994; doi:10.1371/journal.pone.0195224)

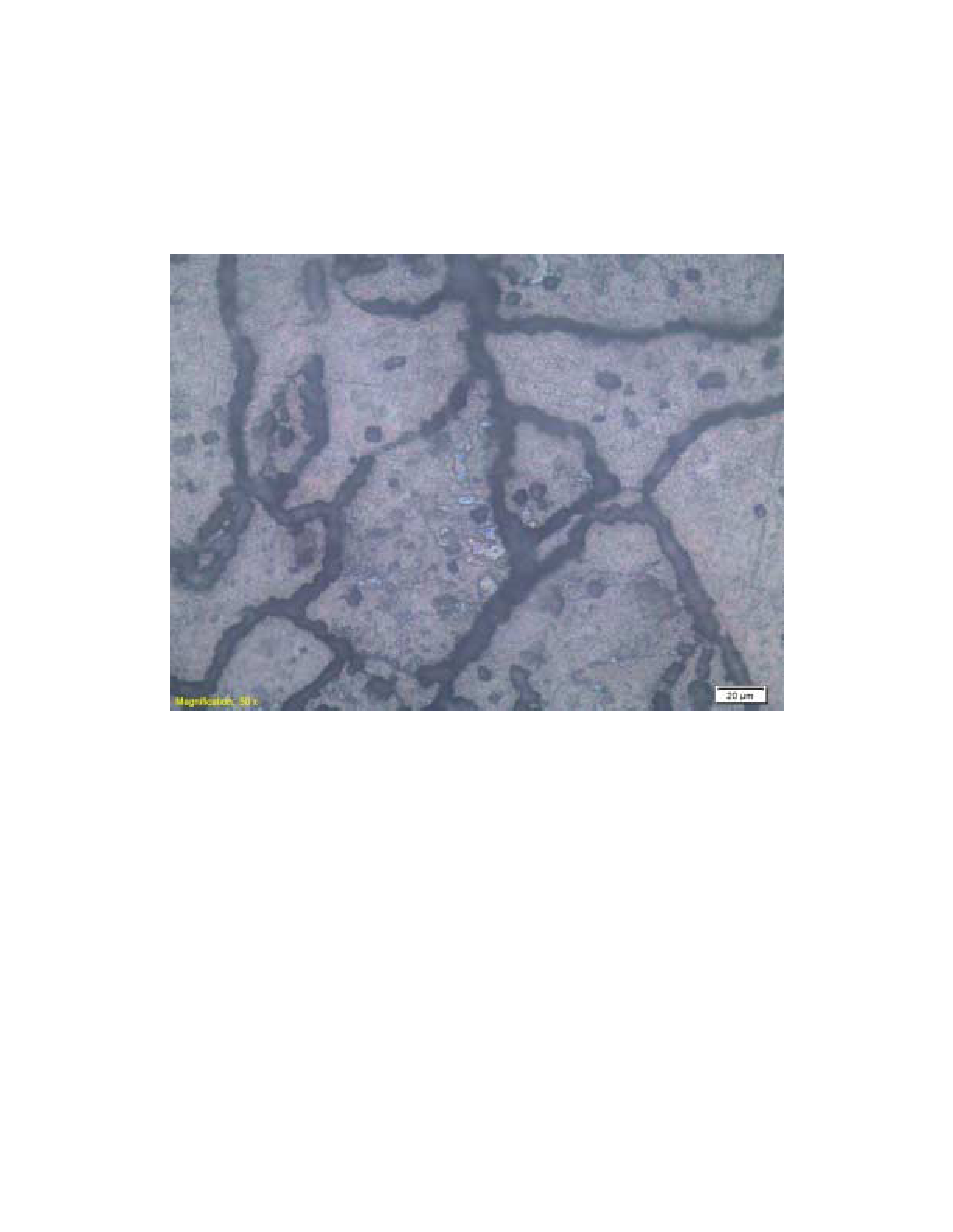

Supplement: S1 Fig — (TIF) [file pone.0195224.s003.tif]

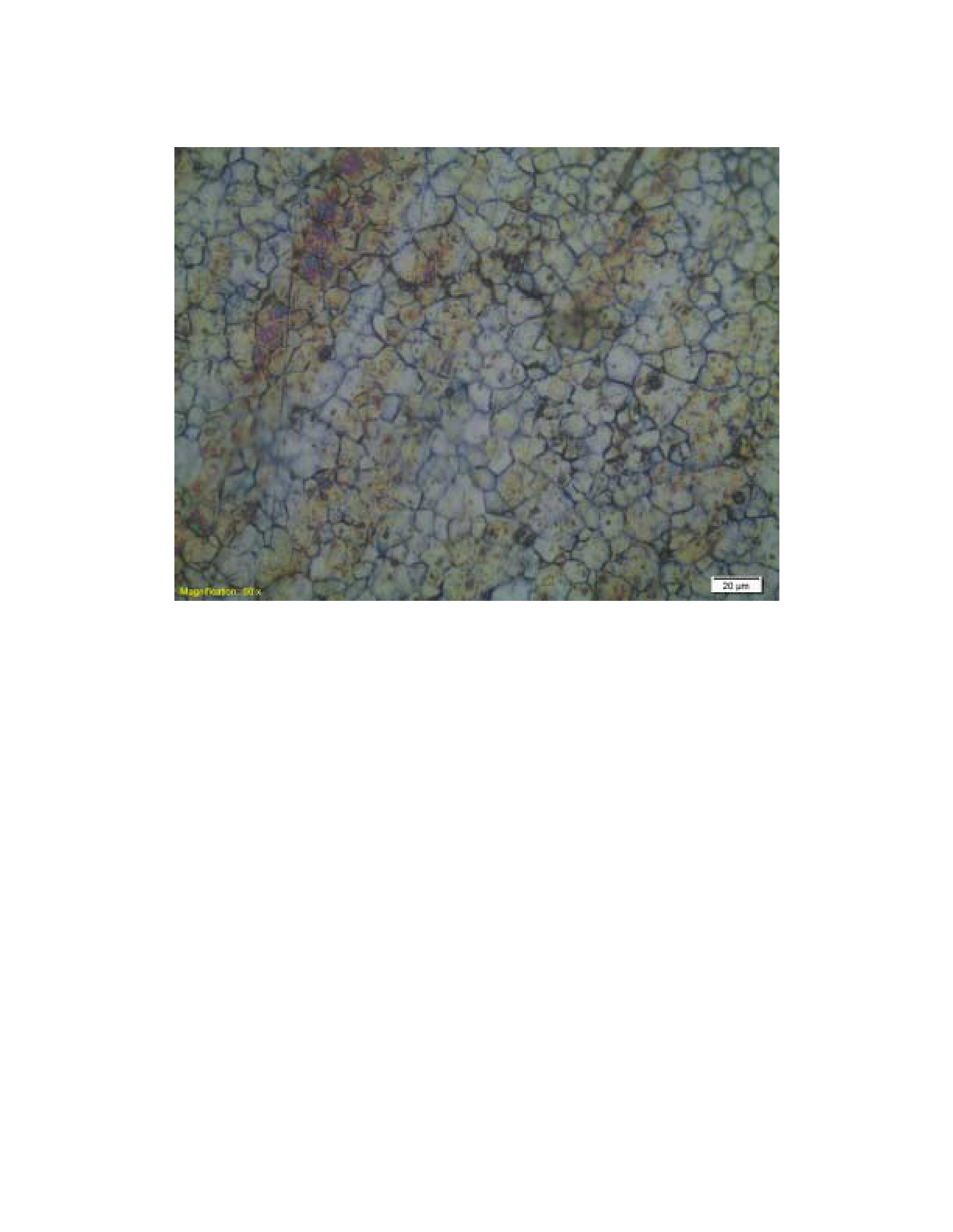

Supplement: S2 Fig — (TIF) [file pone.0195224.s004.tif]

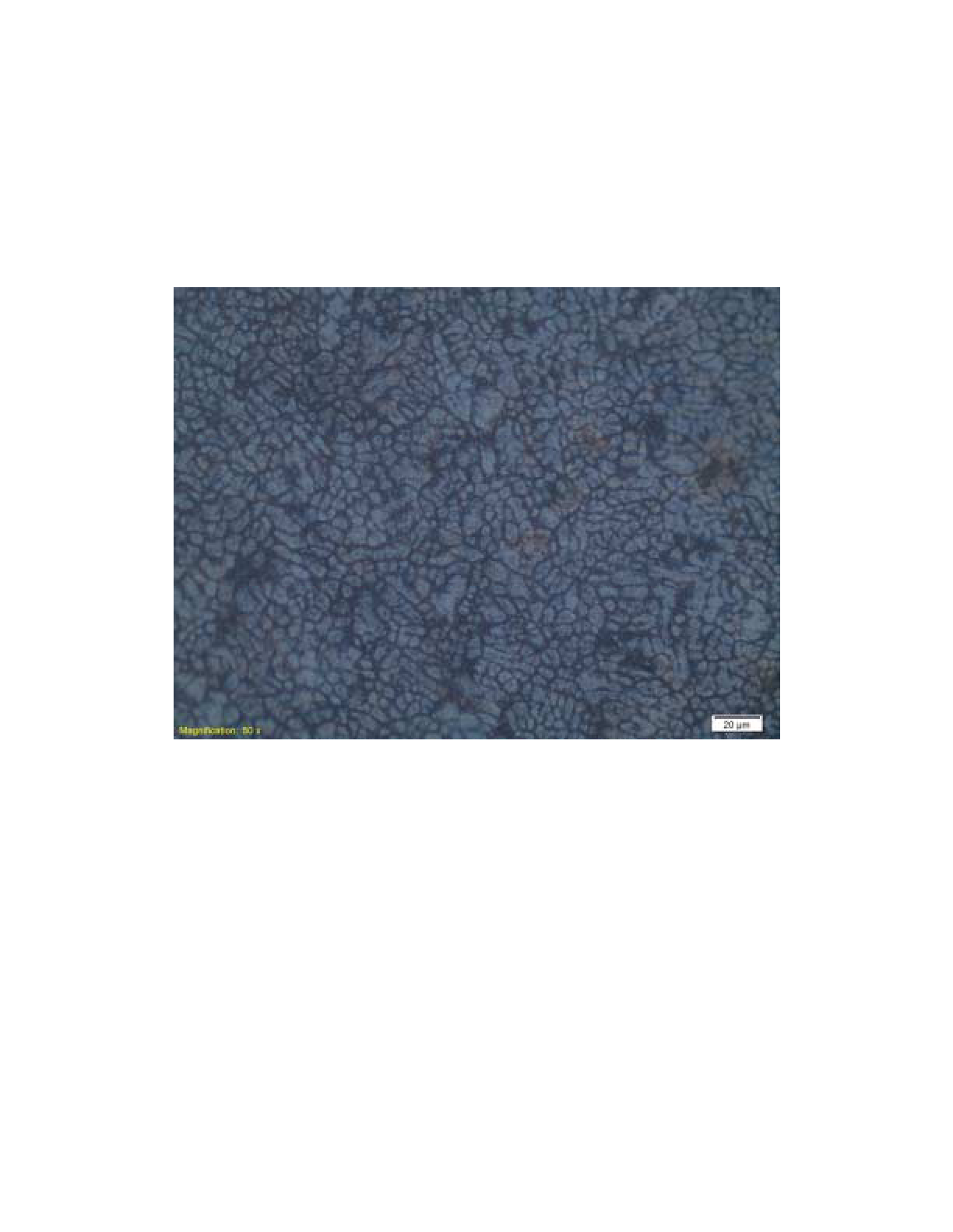

Supplement: S3 Fig — (TIF) [file pone.0195224.s005.tif]

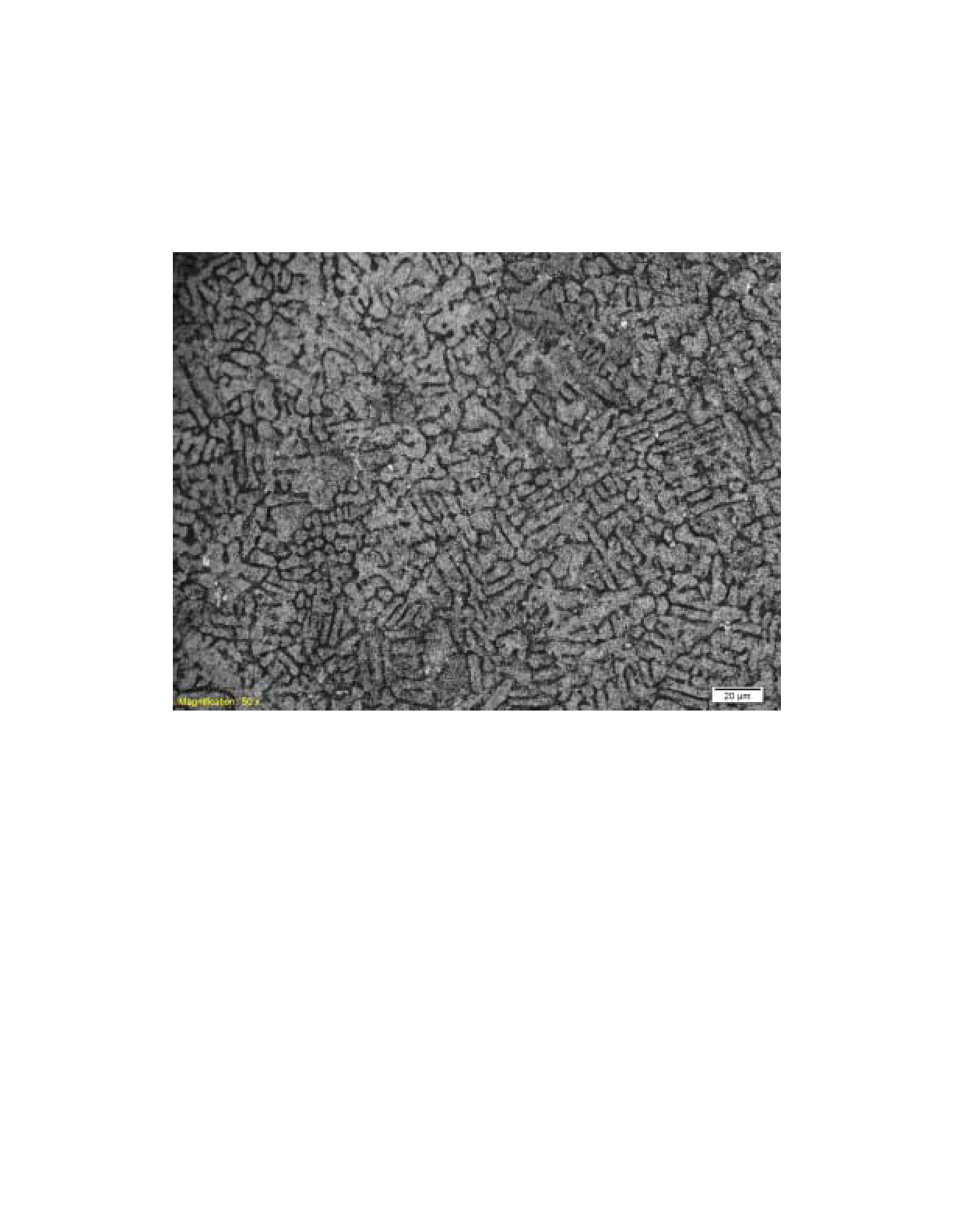

Supplement: S4 Fig — (TIF) [file pone.0195224.s006.tif]

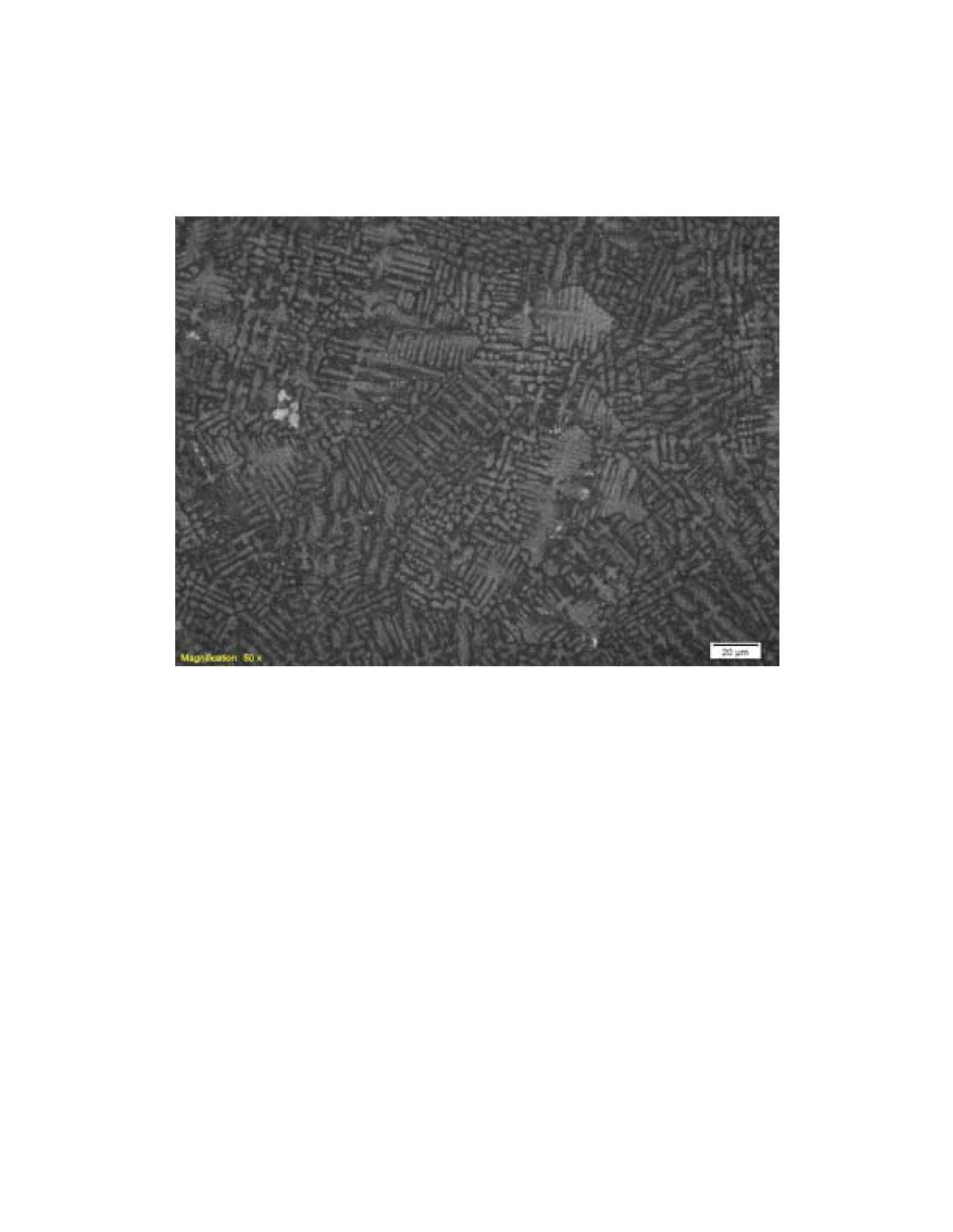

Supplement: S5 Fig — (TIF) [file pone.0195224.s007.tif]
